# Novel resilience in response to revitalisation after exposure to lethal salinity causes differential reproductive success in an extremely plastic organism

**DOI:** 10.7717/peerj.5277

**Published:** 2018-07-31

**Authors:** Mouhammad Shadi Khudr, Samuel Alexander Purkiss, Alice de Sampaio Kalkuhl, Reinmar Hager

**Affiliations:** Evolution and Genomic Sciences, Faculty of Biology, Medicine and Health, The University of Manchester, Manchester, UK

**Keywords:** Resilience, Phenotypic plasticity, Salinity, Survival, Revitalisation, *Daphnia magna*

## Abstract

Phenotypic plasticity is central to an organism’s ability to adapt to variable environmental conditions. For aquatic organisms, exposure to elevated salt levels poses a challenge and organisms may fail to tolerate or survive much higher levels short-term. Here we demonstrate, for the first time, in a laboratory study of *Daphnia magna* that exposure to levels of salinity higher than those previously shown to lead to apparent death (paralysis) can be reversed following a transfer to optimal conditions. We established experimental populations from one clone of *D. magna*, each with five replicates, that were exposed to different short periods of three different lethal levels of salinity (12.27 PSU [45, 60, 90 and 120 min], 18.24 PSU [45, 60 and 90 min] and 24.22 PSU [45, 60 and 90 min]). In all populations, all individuals were paralysed at the end of their exposure, usually classified in the literature as dead. Subsequently, all individuals were transferred to optimal conditions. However, after the transfer, a proportion of the individuals not only came back from the verge of death (i.e. were revitalised), but also showed afterwards differential reproductive success over a period of 20 days, depending on the level and the length of exposure before revitalisation. Both exposure level and time had an overall negative effect on population size that differed across all treatments. Revitalisation occurred within an hour after the transfer to optimal conditions for 18.24 PSU but took 14–16 h for 12.27 PSU. There was no instantaneous revitalisation nor was there any revitalisation after 16 h no matter how long the paralysed *Daphnia* individuals were left in the optimal conditions. Our findings cast new light on resilience in cladocerans and suggest that abrupt environmental change can reveal novel plastic responses to extreme conditions.

## Introduction

Salinisation of freshwater ecosystems is a serious global problem as it affects the composition, abundance and diversity of key zooplankton species (Williams, 2001; Schallenberg, Hall & Burns, 2003; Heine-Fuster et al., 2010). Salinity levels may rise in aquatic ecosystems via natural processes, for example, inverse estuaries, fully and partially mixed coastal waterways (Webster, Atkinson & Radke, 2015) and anthropogenic routes, including salt spreading, mining activities, agricultural and industrial processes (Williams, 2001; Ferguson & Gleeson, 2012; Van Meter & Swan, 2014; Hoover et al., 2017) including effects of global warming (Cañedo-Argüelles et al., 2013).

Phenotypic plasticity, where a given genotype can produce different phenotypes under different environmental conditions (West-Eberhard, 2003), may enable organisms to respond to such changing environments. Most research has focused on either tolerance (an organism’s ability to withstand continuous exposure to a range of conditions under increasing stress), or on resilience (an organism’s adaptability to a range of stressful conditions), (Rodriguez, 2017). However, studies on the potential to recover from what are assumed to be lethal stress levels, for example, extreme salinity, are rare.

### *Daphnia magna* as a crustacean model organism

A key zooplankton organism to study responses to environmental stressors, including salinity, has been *Daphnia magna* because of its importance as a model for biological monitoring (Chapman, Jackson & Krebs, 1996), in toxicological genomics (Shaw et al., 2008), ecological restoration (Li et al., 2013) and resurrection ecology (Burge, Edlund & Frisch, 2017) and for its extreme phenotypic plasticity (Simon et al., 2011). Moreover, *Daphnia* has a relatively short life cycle and reproduce parthenogenetically under optimal conditions (Stollewerk, 2010; Simon et al., 2011).

*Daphnia magna* is commonly found in fresh water environments but some clones have even been discovered living in brackish waters (Schuytema, Nebeker & Stutzman, 1997; Martínez-Jerónimo & Martínez-Jerónimo, 2007). Clones found in fresh water environments, however, may not lose their ability to adapt, long term, to low-level increases in salinity. Furthermore, it has been shown that daphinds (e.g*. D. magna*, *D. longispina* and *D. pulex*) living in habitats of changing quality (e.g. due to rising temperatures, drought, inundation and prolonged salinity exposure) show an ability to resist the effects of escalated salinity stress (Pajunen & Pajunen, 2003).

### Daphnia and salinity stress

On the one hand, daphnid responses to environmental stressors such as salinity have been extensively studied in terms of tolerance (Latta et al., 2012; Garreta-Lara et al., 2016). Previous research has revealed that *Daphnia* can live and reproduce well under saline conditions up to ∼3.98 PSU, and may survive and replicate under short-term exposure to higher salinity levels to a maximum of around 7.46 PSU (Schuytema, Nebeker & Stutzman, 1997). However, acute exposure has a negative effect on metabolic rate (Chen & Stillman, 2012) and can impair reproduction (Ghazy et al., 2009); exposure to high levels of salinity may, as well, lead to immobilisation (paralysis, leading to apparent death; Latta et al., 2012).

On the other hand, *Daphnia* are able to produce durable eggs that can survive harsh conditions and may rest in sediments for long periods to produce viable offspring when the opportunity arises again (Li et al., 2013), which has been interpreted as a bet-hedging strategy against environmental change (Cáceres & Tessier, 2003). Resilience in *Daphnia* has also been studied by investigating the thresholds beyond which the organism usually cannot survive (Gergs et al., 2013). While resilience in *Daphnia* has mostly focussed on the dormant egg stage, few studies have thus far investigated resilience in living daphnids.

For freshwater organisms that inhabit coastal habitats, fluctuating and rising salinity levels present a major challenge because of increased stress, leading to reduced reproduction and impaired development (Albecker & McCoy, 2017). Although cladocerans in general, and *D. magna* in particular, may show tolerance to salinity stress (Schuytema, Nebeker & Stutzman, 1997; Gökçe & Turhan, 2014), increased salinisation not only changes the conditions necessary to maintain normal osmotic pressure and life functions, but can also alter reproductive rates, survivorship rates and population dynamics (Weider & Hebert, 1987; Lignot, Spanings-Pierrot & Charmantier, 2000; Ghazy et al., 2009). Further, elevated levels of stress may lead to extinction of the stressed brackish and freshwater zooplankton from the affected ecosystem as proposed by Gökçe & Turhan (2014) for two species of cladocerans (*Scapholeberis mucronata* and *Simocephalus vetulus*).

*Daphnia magna* is considered a euryhaline species (Smolders, Baillieul & Blust, 2005) and can be found in water containing up to 20% of sea water (Ebert, 2005). It is, nonetheless, sensitive to drastic changes in osmotic pressure and ionic shifts associated with elevated salt levels (Buikema, Geiger & Lee, 1980; Arnér & Koivisto, 1993; Gonçalves et al., 2007).

In our study, we investigated phenotypic plasticity under extreme conditions, and describe a previously unknown resilience phenomenon in *D. magna* in response to acute levels of salinity with different lengths of exposure. The exposed individuals became paralysed (previously assumed dead) characterised by total absence of movement in the appendages except a faint heartbeat and irregular twitching of the internal organs. The paralysed individuals could be revitalised after transfer to a standard optimal medium within 16 h. After investigating such resilience we then sought to establish, in the revitalised individuals, the consequences for subsequent reproduction under standard optimal conditions.

## Materials and Methods

### Study system

A population of genetically identical individuals derived from one clone of *D. magna* was used in this study. This enabled us to control for genotype dependent responses that occur in a genetically variable population and focus our study on salinity level and exposure length responses for a given genotype (Arnér & Koivisto, 1993). Clearly, natural populations would show greater genetic diversity and responses due to different life histories and physiologies. The experimental individuals descended from a culture reared in the laboratory from a sample purchased from Sciento^©^ (Manchester, UK). The clone was maintained in the laboratory for several generations prior to starting the experiment. *Daphnia* were maintained in Aachener Daphnien Medium (ADaM), following Klüttgen et al. (1994), and fed a mix of baker’s yeast *Saccharomyces cerevisiae* and alga *Scenedesmus quadricauda* (1:2 ml, respectively, every other day). The culture and the experimental work took place in a growth chamber (photoperiod 16:8 light:dark, 23 °C, 75% RH) at the Faculty of Biology, Medicine and Health at The University of Manchester.

### Experimental design

We used three different levels of salinity concentrations (12.27, 18.24, 24.22 PSU) based on a pilot study in which paralysed *Daphnia* in concentrations exceeding 9.95 PSU showed the ability to recover from apparent death (paralysis) during a period of 16 h in the optimal medium (ADaM, 0.33 PSU). The levels were also selected to be considerably higher than the levels previously described by Latta et al. (2012) causing apparent death, mimicking adverse brackish conditions for *Daphnia*. Salinity levels were manipulated using sea salt (Sigma-Aldrich, St. Louis, MO, USA) in 300 ml ADaM, in beakers containing six nymphs, randomly selected from the mother clone. There were four different lengths of exposure: 45, 60, 90 and 120 min. With five replicates per salinity level and four exposure times for level 1 (12.27 PSU), three exposure lengths for the other three levels (our pilot work showed that the 120 min exposure leads to complete death and thus was excluded from the treatment), the experimental study population was comprised of 300 individuals. In addition, we recorded data from one beaker of six nymphs in ADaM as a negative control.

After 30 min exposure to any of the three salinity levels, all individuals showed complete paralysis (verified by examining any movement of every individual in the beaker, after careful stirring and under the microscope). Individuals, however, were left until the end of the exposure time in their respective conditions, after which all individuals were transferred from the experimental media to beakers containing 300 ml standard medium (ADaM). One hour after transfer, all individuals were checked for signs of life (motion in any of the appendages), and again at 6, 12, 16 and 24 h after transfer. If individuals did not regain normal or quasi-normal function within 16 h they did not survive and were recorded dead accordingly. Those individuals that did survive (i.e. were able to be revitalised) stayed in the same beaker in the standard medium for 20 days, and population sizes were recorded at d10 and d20. This was done for all treatments with revitalised *Daphnia* (12.27 PSU (45, 60, 90 and 120 min) and 18.24 PSU (45, 60 and 90 min)). Note that no individuals survived the 24.22 PSU treatment and hence no individuals could be revitalised from this treatment. We also recorded the time it took for the first brood to emerge (i.e. the first day any newborn was observed in a given beaker), and the age structure of the population on d10 and d20 by counting the number of juveniles and adults separately.

### Statistical analysis

#### Daphnia revitalisation

We calculated the proportion of the six *Daphnia* individuals that were revitalised within a 16 h period. A generalised linear model (GLM) was applied with a quasi-Poisson family, R package ‘multcomp’ (Hothorn, Bretz & Westfall, 2008). The predictors were: (1) salinity stress (three levels of salinity), (2) exposure time (four levels), (3) all interactions between salinity and exposure time. This was followed by posthoc Tukey’s HSD test, R package ‘lsmeans’ (Lenth, 2016), to analyse pairwise comparisons.

#### Fitness and age-structure of the revitalised Daphnia population

Population size was determined on d10 and d20 (allowing two possible generations under standard conditions). A generalised mixed effect model was used with a Poisson family, R packages ‘lme4’ (Bates et al., 2015) and ‘car’ (Fox & Weisberg, 2011). The predictors were: (1) salinity stress (three levels), (2) exposure time (four levels), (3) all interactions between salinity and exposure time. The census time and the beaker were randomised factors. The number of replicates depended here on the beakers containing any revitalised individuals: Five beakers corresponding to exposure to 12.27 PSU (45, 60, 90 and 120 min), five beakers corresponding to exposure to 18.24 PSU (45 min) and two beakers corresponding to exposure to 18.24 PSU (60 min). For the last treatment, three of the five initial beakers contained no individuals that could be revitalised. All data analyses were conducted using R (R Core Team, 2017).

## Results

Following exposure to the salinity treatments, *Daphnia* were found in a state of paralysis, without noticeable movement in the appendages (Brack et al., 1999), described as ‘death’ by Latta et al. (2012), which occurred at 6.97 PSU for a specialist clone and at 7.96 PSU for a generalist one in their study. However, in our study, we noticed a faint heartbeat and irregular twitching of the internal organs in the paralysed individuals and that, after transfer to the standard medium, the animals could regain some functionality (i.e. they appeared less agile than normal *Daphnia* and their swarming behaviour was abnormal). We were thus able to measure the effects of different levels of salinity experienced for different lengths of time on population growth and reproductive patterns in animals that regained functionality from a state of paralysis (apparent death, see Supplemental Information, Movies S1–S3). We believe that such resilience, given optimal conditions and the ability to recover has not been described to date.

### Revitalisation

The time it took paralysed individuals to regain functionality differed between levels of salinity in an unexpected way. For Salinity Level 1 (12.27 PSU), none of the individuals regained full mobility and apparent vitality within 1h but took between 14 and 16 h post exposure, independent of the exposure time. Surprisingly, individuals exposed to the higher salinity level of 18.24 PSU all showed full mobility and apparent vitality within 1 h, again independent of the length of exposure.

**Figure 1:**
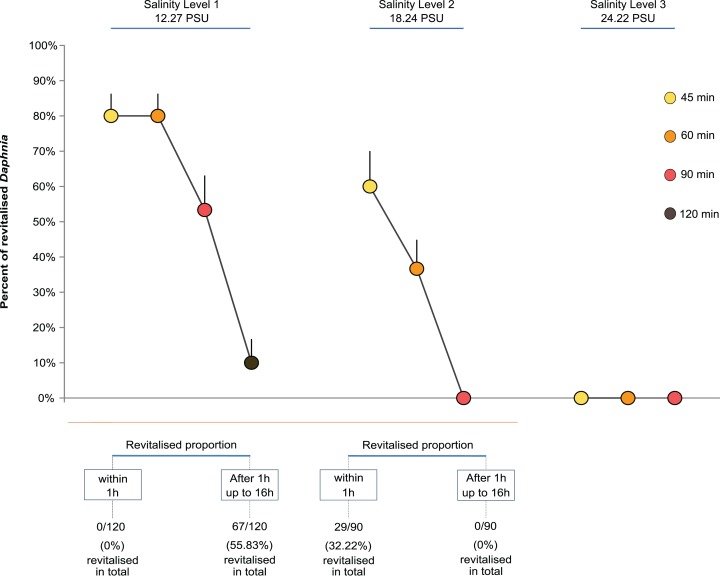
Daphnia revitalisation. Average proportions (±SE) of *Daphnia* revitalised after exposure to different levels and times of acute salinity stress, *n* = 300 *Daphnia* (Level 1, 12.27 PSU, (four exposure times × five replicates (beakers) × six neonates per replicate = 120) + Levels 2, 18.24 PSU, and 3, 24.22 PSU, (three exposure times × five replicates (beakers) × six neonates per replicate = 180)).

Overall, the proportion of revitalised *D. magna* within 16 h declined with higher exposure time and salinity. The GLM showed that both salinity level and exposure time strongly affected the proportion of *Daphnia* that regained full mobility within 16 h (Fig. 1). Both the level of salinity and exposure time had a significant negative effect (GLM, *F*_(2,47)_ = 117.12, *P* < 0.0001, and *F*_(3,44)_ = 34.86, *P* < 0.0001). Further, the interaction between the two factors had a significant negative effect such that higher levels of salinity and longer exposure reduced the proportion of revitalised individuals (*F*_(4,40)_ = 11.31, *P* < 0.0001; Fig. 1). For multiple pairwise comparisons of the different salinity levels and different lengths of exposure, see Supplemental Information Appendix, Table S1.

Figure 1 shows that the percentage of revitalised individuals was higher with shorter exposure time and lower salinity levels. No revitalisation occurred under the highest salinity level (24.22 PSU, *Cf*. sea water ∼34.84 PSU), nor under Salinity Levels 2 and 3 when exposed for longer than 60 min.

### Population dynamics and growth post revitalisation

**Figure 2:**
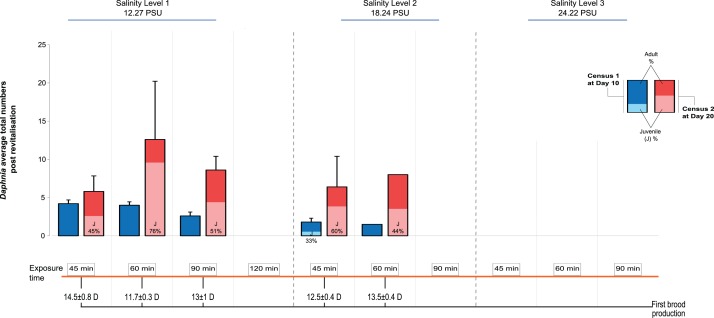
Post-revitalisation *Daphnia* population parameters across treatments. Average total numbers (±SE) of revitalised *Daphnia* on day 10 and day 20 after revitalisation. The total starting population immediately post revitalisation was *n* = 96 revitalised *Daphnia* (Level 1 [45 min (24 individuals of five beakers), 60 min (24 individuals of five beakers), 90 min (16 individuals of five beakers), 120 min (three individuals of two beakers)], Level 2 [45 min (18 individuals of five beakers), 60 min (11 individuals of five beakers), 90 min (zero individuals)]).

We recorded the day of first brood production and population size at d10 and d20 after transfer to the standard medium, distinguishing between adult and juvenile individuals. The first day of brood production showed a surprising pattern among the treatments: In contrast to predictions of a longer time taken with longer periods of exposure and higher levels of salinity (indicative of increased severity, for example, Gonçalves et al., 2007), we found that in Salinity Level 1 those individuals exposed for 60 min were almost 3 days quicker in producing the first brood, after revitalisation, than those exposed for 45 min (11.7 ± 0.3 vs 14.5 days ± 0.8 SE; Fig. 2). By contrast, individuals exposed to Salinity Level 2 took 12.5 ± 0.4 vs 13.5 days ± 0.4 SE, post revitalisation. However, this difference in day of first brood production was statistically not significantly affected by salinity level, exposure time or their interaction. The effects of salinity exposure seen above may be caused by a trigger effect that initiates reproduction when conditions become hospitable again (in optimal conditions following revitalisation). Further, no animals were able to be revitalised from Salinity Level 3 possibly due to more detrimental effects of higher salinity to metabolism, which in turn may impair future reproduction further.

Next, looking at predictors of population size we found that salinity had a significant negative effect (GLMM, *Χ*^2^ = 37.53, d*f* = 1, *P* < 0.0001), as did the length of exposure (*Χ*^2^ = 46.72, d*f* = 3, *P* < 0.0001), as well as the interaction between the two predictors, such that population size decreased with increased salinity level and length of exposure (Fig. 2; *Χ*^2^ = 24.60, d*f* = 2, *P* < 0.0001). For multiple pairwise comparisons of the different salinity levels and different lengths of exposure see Supplemental Information Appendix, Table S2.

Total population size was highest again in the 60 min exposure of Salinity Level 1 (∼12 individuals, significantly larger than in the 45 min exposure group) reflecting the effect of the earlier start of reproduction in this group. For comparison, population size in the control population (starting population size 6) was 35 (5 adults and 30 juveniles) on d10 and 226 on d20 (55 adults and 171 juveniles). Among the treatment populations, none contained any newly produced juveniles at d10, while at d20 the number of juveniles ranged from an average of 2.6 ± 1.5 to 9.6 ± 6.4 SE. Further, the juveniles found at d10 in Salinity Level 2 originated from the original population and thus showed delayed development.

## Discussion

Our study has revealed a novel aspect of phenotypic plasticity in *D. magna* after short exposure to high levels of salinity, which resulted in paralysis (apparent death) after 30 min: we were able to revitalise individuals by transferring animals to the standard medium, which surprisingly took longer (14–16 h) for lower levels of salinity but was almost instantaneous for higher levels. Thus, if the abrupt change was followed by transfer or exposure to hospitable optimal conditions, *D. magna* can show greater resilience to lethal levels of environmental stressors, than previously assumed and continue reproduction, albeit at a lower rate compared to control.

### Salinisation challenge

The salinisation of freshwater has profound economic and environmental costs (Williams, 1999). The salinity content of estuaries as well as water bodies in which *Daphnia* live are drastically affected by several factors including sea-level rise and associated ground water salinisation (Ferguson & Gleeson, 2012; Hoover et al., 2017), storm surges and tidal-seawater intrusion (White & Kaplan, 2017). Further, owing to the mix of rain and seawater, salinity can reach up to 8.68 PSU under droughts and higher temperatures during the dry season (Pajunen & Pajunen, 2003). Moreover, anthropogenic effects of de-icing salt (Van Meter & Swan, 2014) can lead to serious ionic changes due to increased levels of chloride in freshwater (Dugan et al., 2017), with far-reaching effects on aquatic life (Liu & Steiner, 2017) because of salinity changes related to river diversion, dam building and leaching of salts (Zeinoddini, Bakhtiari & Ehteshami, 2015; Verma & Pandey, 2017).

The eventual outcome of continued salinisation is thought to be a global reduction of fresh water biodiversity as its effects are felt at multiple levels (individual, population, community and ecosystem) (Cañedo-Argüelles et al., 2013). Global warming is predicted to increase the incidence of inundation in freshwater ecosystems as sea levels rise, and extreme weather events such as droughts and storm surges occur more often (Jeppesen et al., 2015). Thus, the environment for daphnids can be unpredictable with wide fluctuations in salinity level affecting their fitness, development and survival (Cowgill & Milazzo, 1991; Arnér & Koivisto, 1993). Daphnid populations found near seawater may experience inflow from other aquatic habitats of more brackish content that may result in mass mortality. Subsequent rainfall could reduce levels of salinity rapidly again, providing a scenario for recovery and perhaps adaptation (resilience) to such fluctuating conditions.

### Revitalisation

*Daphnia* has evolved to cope with life in aquatic habitats with relatively low osmotic pressure. However, salinity conditions are often not optimal or stable and thus the permeable soft body of daphnids will always be osmotically challenged, by numerous natural and human-induced factors (Williams, 1999; Ghazy et al., 2009; Jeppesen et al., 2015). Despite being able to cope with higher levels of salinity than found in freshwater (Schuytema, Nebeker & Stutzman, 1997), in face of increased salinisation, *Daphnia* have four possibilities: avoid, disperse, adapt or perish (Arnér & Koivisto, 1993; Nielsen et al., 2003; Pajunen & Pajunen, 2003). Indeed, acute exposure to extreme levels of salinity exceeding maximum levels that can be tolerated leads to complete paralysis in ephemeral habitats and will severely disturb the osmoregulatory capacity and metabolic rates in *Daphnia* (Hall & Burns, 2001). This may be the reason for the quasi-normal reproductive patterns seen in the revitalised animals in our experiment. From a physiological point of view, the salinity level might have been the most relevant factor affecting the revitalisation due to the fact that intensified stress corresponded with less resilience (Arnér & Koivisto, 1993; Gonçalves et al., 2007; Gergs et al., 2013). The point of ‘no return’ from paralysis was, nevertheless, between 18.24 PSU for 120 min, and 24.22 PSU (Fig. 1).

### Osmoregulation and osmoconformance

*Daphnia* survival is dependent on its halotolerance and ability to adapt to external ionic challenge, salinity level and haemolymph osmolality, where *Daphnia* remarkably might switch between two context-dependent osmoregulatory strategies: osmoregulation and osmoconformity (Weider & Hebert, 1987; Heine-Fuster et al., 2010). Osmoregulation is a typical fresh-water organism response, where the organism keeps haemolymph osmolality constant; a strategy that works up to a salinity level of 4.98 PSU. By contrast, osmoconformity is a typical response of marine and brackish-water organisms. With salinity levels surpassing 4.98 PSU, *Daphnia magna* will lower the osmotic gradient between haemolymph and external saline environments that may enable the organism to tolerate a broad range of salinities (Weider & Hebert, 1987; Heine-Fuster et al., 2010).

Salinity of ponds and coastal lakes with varying distances from seashores can change greatly and abruptly. This presents a major energetically costly ionic challenge for *Daphnia* to osmoregualte (Weider & Hebert, 1987), which may result in impairment of ionic exchange and the mechanisms of osmoregulation under elevated levels of salinity (Lignot, Spanings-Pierrot & Charmantier, 2000). For instance, the osmolality measurement run by Weider & Hebert (1987) on *D. pulex* from bluff ponds near Hudson Bay, Canada, revealed that these hyperconformers could not survive a salinity level of 4.98 PSU for more than a few hours. Thus, periodic induced tidal fluctuations and intermittent saline intrusions can have detrimental effects on osmoregulation in *Daphnia* (Lignot, Spanings-Pierrot & Charmantier, 2000; Hall & Burns, 2001; Schallenberg, Hall & Burns, 2003).

In our study, *Daphnia* went into paralysis at levels that far exceed 4.98 PSU and we can thus speculate that the sudden change from ADaM (optimal conditions of 0.33 PSU) to 12.27 PSU and 18.24 PSU made it impossible to adapt and regulate haemolymph concentrations to match the extreme osmotic pressure. However, once in ADaM, the individuals may have been able to restore haemostasis. Osmoregulation in response to salinity stress has previously been observed in marine crustaceans (Charmantier & Charmantier-Daures, 1991). Marine crustaceans are exposed to salinity fluxes through tides and have highly varied responses to these stressors (Huni & Aravindan, 1985); for example, growth and morphology are shown to be affected by such environmental stressors (López et al., 2010). By contrast, halophylic *D. exilis* shows a hyper-osmoconformer physiological response to salinity stress as well as a delayed maturity age and reduced fertility. The response is achieved by maintaining a positive osmolality difference with the environment. *D. pulex* also shows hyperosmotic responses (Heine-Fuster et al., 2010). Changes in osmoregulation in *D. magna* are thought to be linked to the epithelial cells of the gill as these cells are required for osmoregulation (Kikuchi, 1983), necessary for ion uptake and transport (Bianchini & Wood, 2008).

In sum, there is considerable evidence for the ability to adapt to salinity stress in daphnids. This ability to adapt has significant ecoevolutionary implications due to the role of *Daphnia*, as zooplankton, in aquatic food webs (Coldsnow et al., 2017; Liu & Steiner, 2017). Tolerating strong fluctuations in salinity requires a well-adapted osmoregulatory system (Gilles & Péqueux, 1983; Weider & Hebert, 1987). However, coming back from the point of no return (apparent death) is an attribute of resilience that goes beyond what is known for tolerance and may require compensatory osmoregulatory mechanisms that may explain the successful revitalisation of *Daphnia* in this study. Future work now needs to establish the physiochemical properties of resilience and recoverability from exposure to lethal levels of salinity stress.

### Reproductive success after revitalisation

We observed two distinctive reproductive patterns whereby the first brood was produced earlier when exposed longer to the stressor compared with a shorter exposure. Although the difference in exposure was only 15 min, the difference in number of days until first brood production was almost 3 days, or 25%, earlier. The difference in number of adults between the d10 and d20 counts, may be due to adult mortality and impaired development caused by the salinity exposure. However, we note that the treatment effect on the production of first brood was statistically non-significant. These results are surprising since we predicted that the higher the stress the greater the reduction in fitness (Arnér & Koivisto, 1993; Gonçalves et al., 2007), which may be associated with different reproductive modes (Arnér & Koivisto, 1993), that is, it would take longer to produce the first brood, and population size increase would be at a lower rate. Our results suggest that different rates of reproduction can be triggered. The higher reproductive rate and shorter time to first brood following revitalisation from paralysis were associated with revitalisation after longer exposure to salinity, which may be similar to parity variation seen in other taxa (Schaffer, 1974; Stearns, 1976; Zeineddine & Jansen, 2009). Note that under more predictable environments large progeny is expected to be favoured because they increase the chances of success especially after major failure (Stearns, 1976). Following recovery from trauma and revitalisation from paralysis, resilience followed by bouts of reproduction (including parity) can be to the benefit of the organism and may be a product of phenotypic plasticity (Hughes & Simons, 2014; Hughes, 2017) in face of ecological challenge, thereby increasing the chances of population survival (Stearns, 1976; Harney, 2013).

In our study, immature, clonal *Daphnia* (genetically identical) underwent severe and hostile conditions that led to their death or to paralysis. The revitalised *Daphnia* afterwards displayed a noticeable delay in maturation, that is, the delay in the production of the first brood. Variation in the maturation threshold for body size has been proposed to depend on environmental and transgenerational effects, leading to fitness consequences (Harney, 2013). For example *D. magna* genotypes with a higher maturation threshold have been demonstrated to have a lower intrinsic rate of population increase (Harney, 2013).

## Conclusion

Our study mimics the scenarios where a population of *D. magna* may experience an abrupt change in salinity level in its habitat, for example, due to sea intrusion and/or inflow from other brackish water sources. In this scenario, subsequent rainfall could provide the conditions sufficient to reverse paralysis as simulated in our study by the transfer of the paralysed individuals to their optimal medium that was followed by differential reproductive success. Clearly, our findings reveal a novel aspect of phenotypic plasticity in *D. magna*, a phenomenon that may well be seen in other cladocerans. Understanding the ecological impacts of resilience following salinity trauma in such ecosystems remains unknown and thus merits further research. Future work needs to elucidate the underlying mechanisms and whether these responses are limited to salinity as a stressor or represent a more universal stress response.

## Supplemental Information

10.7717/peerj.5277/supp-1 Supplemental Information 1 Supportive movies and complemntary statistics. **Movie S1. *Daphnia magna* under optimal condition.** This movie is available via the Figshare repository via the URL <https://figshare.com/s/22543250efd9082173e4>
**Movie S2. *Daphnia magna* in paralysis 1.** This movie is available via the Figshare repository via the URL <https://figshare.com/s/75558515ede3561bbb9b>
**Movie S3. *Daphnia magna* in paralysis 2.** This movie is available via the Figshare repository via the URL <https://figshare.com/s/19304f2e6f454fa55934>
**Table S1. Further pairwise comparisons, revitalisation of *Daphnia*.** A posthoc Tukey test was run following the GLM testing *Daphnia*’s revitalisation (described in the main text) to provide further multiple pairwise comparisons of the revitalisation under different salinity levels (12.27 PSU, 18.24 PSU, 24.22 PSU) and exposure times (45 min, 60 min, 90 min, 120 min). Only significant results are shown. See main text methods and results for further details.
**Table S2. Further pairwise comparisons, reproductive success of *Daphnia* post revitalisation**. A posthoc Tukey test was run following the GLMM (described in the main text) testing *Daphnia*’s differential reproduction after revitalisation to provide further multiple pairwise comparisons under different salinity levels (12.27 PSU, 18.24 PSU) and exposure times (45 min, 60 min, 90 min, 120 min). Only significant and marginally significant results are shown. See main text methods and results for further details.

## Additional Information and Declarations

### Data Availability

The following information was supplied regarding data availability: Figshare: DOI 10.6084/m9.figshare.5709715.
