# Supplementary material for: Novel resilience in response to revitalisation after exposure to lethal salinity causes differential reproductive success in an extremely plastic organism
Source: PeerJ. 2018 Jul 31;6:e5277. doi: 10.7717/peerj.5277 (PMC6074775; doi:10.7717/peerj.5277)
Supplement: Supplemental Information 1 — Movie S1. Daphnia magna under optimal condition. This movie is available via the Figshare repository via the URL <https://figshare.com/s/22543250efd9082173e4> Movie S2. Daphnia magna in paralysis 1. This movie is available via the Figshare repository via the URL <https://figshare.com/s/75558515ede3561bbb9b> Movie S3. Daphnia magna in paralysis 2. This movie is available via the Figshare repository via the URL <https://figshare.com/s/19304f2e6f454fa55934> Table S1. Further pairwise comparisons, revitalisation of Daphnia. A posthoc Tukey test was run following the GLM testing Daphnia’s revitalisation (described in the main text) to provide further multiple pairwise comparisons of the revitalisation under different salinity levels (12.27 PSU, 18.24 PSU, 24.22 PSU) and exposure times (45 min, 60 min, 90 min, 120 min). Only significant results are shown. See main text methods and results for further details. Table S2. Further pairwise comparisons, reproductive success of Daphnia post revitalisation. A posthoc Tukey test was run following the GLMM (described in the main text) testing Daphnia’s differential reproduction after revitalisation to provide further multiple pairwise comparisons under different salinity levels (12.27 PSU, 18.24 PSU) and exposure times (45 min, 60 min, 90 min, 120 min). Only significant and marginally significant results are shown. See main text methods and results for further details. [file peerj-06-5277-s001.doc]

**Novel resilience in response to revitalisation after exposure to lethal salinity causes differential reproductive success in an extremely plastic organism**

Mouhammad Shadi Khudr*, Samuel Alexander Purkiss*, Alice de Sampaio Kalkuhl, Reinmar Hager

*Equal contribution

**Supplemental information**

**Supportive movies**

**Movie S1. *Daphnia magna* under optimal condition.** This movie is available via the Figshare repository via the URL

< <https://figshare.com/s/22543250efd9082173e4> >

**Movie S2. *Daphnia magna* in paralysis 1.** This movie is available via the Figshare repository via the URL

< <https://figshare.com/s/75558515ede3561bbb9b> >

**Movie S3. *Daphnia magna* in paralysis 2.** This movie is available via the Figshare repository via the URL

< <https://figshare.com/s/19304f2e6f454fa55934> >

**Supportive statistics (multiple comparisons)**

**Table S1. Further pairwise comparisons, revitalisation of *Daphnia*.** A posthoc Tukey test was run following the GLM testing *Daphnia*’s revitalisation (described in the main text) to provide further multiple pairwise comparisons of the revitalisation under different salinity levels (12.27 PSU, 18.24 PSU, 24.22 PSU) and exposure times (45 min, 60 min, 90 min, 120 min). Only significant results are shown. See main text methods and results for further details.

|  | |  |  |  |  |  |  |  |
| --- | --- | --- | --- | --- | --- | --- | --- | --- |
| contrast p.value | | | | | | | | |
| 12.33,45 - 18.33,60 0.0084 | | | | | | | | |
| 12.33,45 - 12.33,120 <.0001 | | | | | | | | |
| 12.33,60 - 12.33,120 <.0001 | | | | | | | | |
| 12.33,60 - 18.33,60 0.0084 | | | | | | | | |
| 12.33,90 - 12.33,120 0.0002 | | | | | | | | |
| 18.33,45 - 12.33,120 <.0001 | | | | | | | | |
| 18.33,60 - 12.33,120 0.0216 | | | | | | | | |
|  |  |  |  |  |  |  |  |  |
|  | | | | | | |  |  |

**Table S2. Further pairwise comparisons, reproductive success of *Daphnia* post revitalisation.** A posthoc Tukey test was run following the GLMM (described in the main text) testing *Daphnia*’s differential reproduction after revitalisation to provide further multiple pairwise comparisons under different salinity levels (12.27 PSU, 18.24 PSU) and exposure times (45 min, 60 min, 90 min, 120 min). Only significant and marginally significant results are shown. See main text methods and results for further details.

|  |  |  |  |  |  |  |  |
| --- | --- | --- | --- | --- | --- | --- | --- |
| contrast p.value | | | | | | | |
| 12.33,45 - 12.33,120 <.0001 | | | | | | | |
| 12.33,60 - 12.33,120 <.0001 | | | | | | | |
| 12.33,90 - 12.33,120 <.0001 | | | | | | | |
| 12.33,45 - 18.33,60 0.0270 | | | | | | | |
| 12.33,45 - 18.33,90 <.0001 | | | | | | | |
| 12.33,60 - 18.33,60 <.0001 | | | | | | | |
| 18.33,45 - 12.33,60 0.0124 | | | | | | | |
| 12.33,60 - 18.33,90 <.0001 | | | | | | | |
| 12.33,90 - 18.33,90 <.0001 | | | | | | | |
| 18.33,60 - 12.33,90 0.0051 | | | | | | | |
| 18.33,60 - 12.33,120 0.0689 | | | | | | | |
| 18.33,45 - 12.33,120 0.0001 | | | | | | | |
| 18.33,45 - 18.33,90 0.0001 | | | | | | | |
| 18.33,60 - 18.33,90 0.0689 | | | | | | | |
